# Trained Immunity Enhances Human Monocyte Function in Aging and Sepsis

**DOI:** 10.3389/fimmu.2022.872652

**Published:** 2022-05-25

**Authors:** P. Spencer Gill, Tammy R. Ozment, Nicole H. Lewis, Edward R. Sherwood, David L. Williams

**Affiliations:** ^1^Department of Surgery, James H. Quillen College of Medicine, East Tennessee State University, Johnson City, TN, United States; ^2^Center for Inflammation, Infectious Disease, and Immunity, Quillen College of Medicine, East Tennessee State University, Johnson City, TN, United States; ^3^Department of Medical Education, Quillen College of Medicine, East Tennessee State University, Johnson City, TN, United States; ^4^Department of Pathology, Microbiology, and Immunology, Vanderbilt University Medical Center, Nashville, TN, United States; ^5^Department of Anesthesiology, Vanderbilt University Medical Center, Nashville, TN, United States

**Keywords:** trained immunity, aging, immunosenescence, innate immunity, monocytes

## Abstract

Aging plays a critical role in the incidence and severity of infection, with age emerging as an independent predictor of mortality in sepsis. Trained immunity reprograms immunocytes to respond more rapidly and effectively to pathogens and serves as a potential approach to improve immune function in aging and/or sepsis. However, there is very little data on trained immunity in the aging immune system or in the presence of sepsis. We examined the impact of β-glucan induced innate immune training on monocytes from aging healthy humans (>60 years old) as well as sepsis patients. We observed increased metabolic capacity, upregulated cytokine secretion, increased H3K27 acetylation, and upregulation of crucial intracellular signaling pathways in trained monocytes from healthy aging subjects. The response to trained immunity in healthy aging monocytes was equivalent to the response of monocytes from younger, *i.e.*, 18 – 59 years, individuals. Additionally, we found that trained immunity induced a unique expression pattern of cell surface markers in monocytes that was consistent across age groups. Trained monocytes from sepsis patients also displayed enhanced metabolic capacity and increased cytokine production. These results indicate that immune training can be induced in aging monocytes as well as monocytes from critically ill sepsis patients.

## Introduction

It has been estimated that over the next thirty years the number of individuals >60 years of age will more than double, increasing by over a billion; and the number of individuals over age 80 may increase by as much as 300 million (1). This dramatic increase in the number of older individuals will result in a significant increase in age related diseases. As humans age, our immune system becomes progressively weaker through a process called immune senescence (1). This age-related decrease in immune function increases susceptibility to infection and sepsis (1–4).

Sepsis is the leading cause of death in non-cardiac intensive care units (ICU) and accounts for 40% of ICU expenditures (5). Patients that survive sepsis have long-term physical and cognitive disabilities and are frequently re-admitted to the hospital with recurrent infections (6–9). Over the past two decades there has been an increased incidence of sepsis (10) and this trend is likely to continue due to our aging population, increased use of immunosuppressive drugs and invasive procedures, and the emergence of antibiotic resistant opportunistic pathogens (5). Age has emerged as an independent predictor of morbidity and mortality in sepsis (11, 12). Indeed, 60% of sepsis cases occur in patients over 65 years of age (11, 13). It is generally accepted that age related immune senescence increases susceptibility to infection and sepsis (11, 13), which raises the question of whether it is possible to modulate the aging immune system to improve resistance to infection. One possible approach to enhancing immune function during aging is innate immune training (14, 15).

There is a substantial literature demonstrating that the innate immune system can be trained to respond more rapidly and effectively to infection (14, 16–19). This phenomenon is referred to as “trained immunity” or “innate immune memory” (19). Trained immunity is characterized by metabolic and epigenetic reprogramming in leukocytes in conjunction with enhanced antimicrobial functions (18). However, there is very limited information available on the effect of trained immunity in aging and/or sepsis. In 2011, Wardhana and colleagues reported that BCG vaccination prevented respiratory infections and improved cytokine production in individuals 60 – 75 years of age (20). Additionally, a 2020 clinical trial found that BCG vaccination increased protection from infection in individuals over 65 years old (21). While trained immunity increases inflammatory cytokine production upon restiumulation, interestingly Koeken and colleagues found that BCG vaccination reduces systemic inflammation (22). It is now known that BCG, a potent immune training agent, induces the immune trained phenotype in humans (17, 23), thus it is reasonable to speculate that the effect of BCG on respiratory infections in aging subjects may be mediated, in part, by trained immunity. In this study, we examined innate immune training in monocytes isolated from healthy aging subjects and compared and contrasted their response to immune training with monocytes isolated from younger healthy individuals. We also examined innate immune training in monocytes derived from patients diagnosed with sepsis. We found that trained immunity increases metabolism and functionality of monocytes isolated from healthy aging subjects as well as in sepsis patients.

## Materials and Methods

### Isolation of Human Monocytes From Healthy Subjects

Monocytes have been extensively employed in the study of trained immunity (14, 24). Whole blood containing EDTA was purchased from BioIVT (Gray, Tennessee). The blood samples were de–identified except for age, gender, and race. BioIVT provided blood that was collected from healthy individuals in 3 age groups, *i.e.* 20–30, 31–59, >60 years of age. There were 35 individuals in each age group and a total of 105 individuals in the study. The age range for the entire cohort was 20–71 years. A detailed description of the subjects is provided in **Table 1**. Monocytes were isolated from whole blood using immunomagnetic negative selection with the EasySep™ Direct Human Monocyte Isolation Kit (StemCell #19669). Monocyte purity was assessed by flow cytometry. This method yields CD14+ monocyte purity of up to 97.1%.

**Table 1:** Age, sex, and race breakdowns for healthy human subjects in each age group.

Age Group	Number of Individuals	Mean Age (stdev)	Female (%)	Male(%)	Caucasian (%)	Black (%)	Hispanic (%)	Asian(%)
20–30	35	25.4 (3.0)	16 (45.7%)	19 (54.3%)	29 (82.9%)	2 (5.71)	4 (11.4%)	0 (0%)
31–59	35	41.8 (8.3)	13 (37.1%)	22 (62.9%)	31 (88.6%)	3 (8.6%)	1 (2.9%)	0 (0%)
60+	35	65 (3.1)	23 (65.7%)	12 (34.3%)	33 (94.3%)	0 (0%)	0 (0%)	2 (5.7%)
Combined	105	44.1 (17.2)	52 (49.5%)	53 (50.5%)	93 (88.57%)	5 (4.8%)	5 (4.8%)	2 (1.9%)

### Isolation of Monocytes From Sepsis Patients

Peripheral blood was obtained from sepsis patients at our participating hospital, Johnson City Medical Center, following informed consent (IRB study # 098–98s–MSHA). All patients met the Sepsis–3 criteria (25). A detailed description of the included sepsis patients is provided in **Table 2**. Of the 5 patients included in the study, 2 were female and 3 were male. The patients ranged in age from 23–78 with the average age being 58.6. The average SOFA score of the cohort was 1.6, and the average Apache II score was 8.4. Peripheral blood was collected in BD Vacutainer^®^ CPT™ (BD #362760) tubes. Monocytes were isolated from total mononuclear cells using the EasySep™ Human Monocyte Isolation Kit (StemCell #19359) following manufacturer instructions.

**Table 2:** Description of sepsis patients.

Patient	Age	Race	Sex	Type of Infection	SOFA	APACHE
1	48	White	Male	Staphylococcus aureus	0	Apache II score: 6 points (3% postoperative mortality, 8% nonoperative mortality)
2	68	White	Female	Staphylococcus aureus	2	Apache II Score: 9 points (3% estimated postoperative mortality, 8% estimated nonoperative mortality)
3	76	White	Male	Citrobacter species	3	Apache II Score: 13 points (7% est postoperative mortality)
4	78	White	Male	Enterobacteriaceae, Klebsiella species	3	Apache II Score: 8 points
5	23	White	Female	Sepsis due to Enterococcus faecalis (12 days prior to consent)	0	Apache II Score: 6 points

### Immune Training Protocol

**Figure 1:**
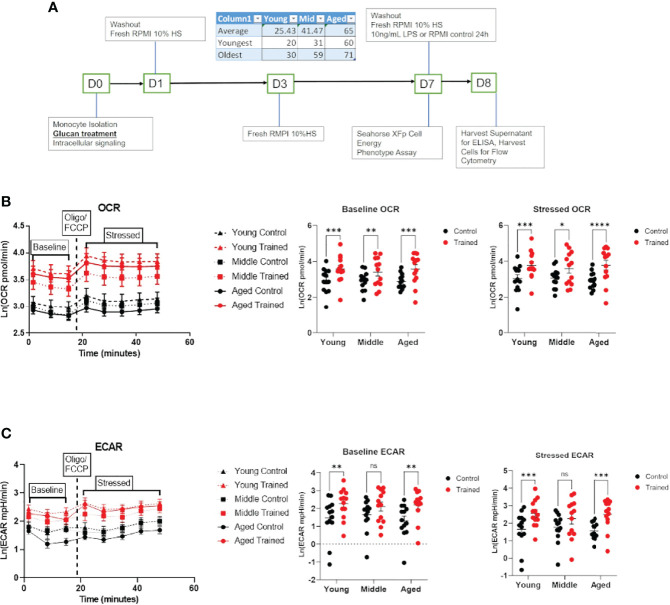
Immune training increases overall metabolic rate of monocytes across all age groups. **(A)** *Ex vivo* trained immunity protocol. **(B)** Baseline and stressed oxygen consumption rate (OCR) 7 days after β–glucan or PBS treatment. **(C)** Baseline extracellular acidification rate (ECAR) and stressed ECAR 7 days after β–glucan or PBS treatment. Mixed effect model and Šídák’s multiple comparisons test (*P ≤ 0.05, **P ≤ 0.01, ***P ≤ 0.001, ****P ≤ 0.0001) were used to compare **(B, C)** (N=13). ns, not significant.

Monocytes were adjusted to 5 x 10^5^ cells/mL in serum–free RPMI 1640 with 1% Antibiotic–Antimycotic (Gibco 15240–062) and treated with 10 μg/mL β–glucan, isolated from *Candida albicans* as previously described (26). The *C. albicans* β–glucan employed in this study was >95% pure. The β–glucan was depyrogenated to remove any residual endotoxin, and it was sterile. The lack of endotoxin in the β–glucan preparation was established with the HEK–Blue LPS Detection kit 2 (Invivogen). Sterility of the β–glucan was confirmed by microbiological testing. Isovolumetric PBS was added to monocyte cultures as a control. Monocytes from the same individual were divided between both treatments. Glucan was washed out after 24h (**Figure 1A**), and media was replaced with RPMI 1640 with 1% Antibiotic–Antimycotic and supplemented with 10% human serum. The media was replenished at day 3. On day 7, the media volume was reduced, and cells were treated with 10 ng/mL LPS (*Escherichia coli* O55:B5, Sigma L6529–1MG) or isovolumetric PBS to serve as baseline control for 24 hours. On day 8, the supernatants were harvested for cytokine analysis, and the cells were stained with antibodies for cell surface markers to be analyzed by flow cytometry.

### Metabolic Assays

Metabolic analysis of isolated monocytes was performed using the Agilent Seahorse XFp Analyzer (Santa Clara, CA). Monocytes were seeded at 1 x 10^5^/well and trained using the protocol defined above. The XFp Cell Energy Phenotype test template was used according to the manufacturer’s specifications. Oxygen consumption rate (OCR) was measured to approximate respiration, and extracellular acidification rate (ECAR) was measured to approximate glycolysis. Baseline OCR/ECAR was calculated by averaging the first three measurements prior to oligomycin/FCCP injection. Stressed OCR/ECAR was calculated by averaging the five measurements taken after oligomycin/FCCP injection. Final in well concentrations of oligomycin (Sigma 495455–10MG) and FCCP (Sigma C2920–10MG) were both 1µM.

### Cytokine Measurements

TNF–α and IL–6 concentrations in conditioned media were analyzed by ELISA (TNA–α: BioLegend 430204, IL–6: BioLegend 430504). Cell culture supernatants were collected and stored at –80°C prior to analysis. ELISAs were performed following manufacturer instructions.

### Measurement of Cell Surface Markers by Flow Cytometry

Trained and control cells were harvested, blocked using block buffer (PBS, 5% rabbit serum, 0.5% goat serum albumin, 5 mM EDTA, 0.1% NaN_3_) and stained for flow cytometry using the following mouse anti–human antibodies: CD14 FITC, Clone TÜK4 (Bio–Rad MCA1568F), HLA–DR PerCP–Cy5.5, Clone G56–6 (BD Biosciences 560652), CD123 PE, Clone 9F5 (BD Biosciences 555644), CD11b/Mac–1 PE–Cy7, Clone ICRF44 (BD Biosciences 557743), CD40 APC, Clone 5C3 (BD Biosciences 555591), CD16 Pacific Blue™, Clone 3G8 (BD Biosciences 558122). All flow cytometry experiments were performed on a BD LSRFortessa flow cytometer. Cells were gated by forward scatter, side scatter, and CD14 positivity (**Figure S1B**). Cell gating was adjusted to account for glucan particles that remained from the initial treatment, given that some residual particles remain after multiple wash steps (**Figure S1A**). Mean fluorescence of isotype controls was subtracted from sample mean fluorescence to normalize between experimental replicates.

### Intracellular Signaling Studies

Trained and control monocytes were adjusted to a density of 5 x 10^6^ cells/mL in 12 x75 mm tubes in RPMI 1640. Cells were treated with 10 μg/mL β–Glucan or vehicle for 1 or 2 hours at 37°C. Cells were fixed using BD Cytofix (BD Biosciences 554655), permeabilized with BD Phosflow Perm Buffer III (BD Biosciences 558050) and stained for 1 hr. with the following antibodies: Alexa Fluor^®^ 488 Mouse anti–Akt1, Clone 55/PKBa/Akt (BD Biosciences 560048, Alexa Fluor^®^ 647 Mouse anti–Akt (pS473), Clone M89–61 (BD Biosciences 560343), PE Mouse Anti–mTOR (pS2448), Clone O21–404 (BD Biosciences 563489), PerCP/Cyanine5.5 anti–ZAP70 Phospho (Tyr319)/Syk Phospho (Tyr352) Antibody, Clone 1503310 (Biolegend 683710). Cells were gated for CD14 positivity on a BD LSRFortessa flow cytometer (**Figure S2**). Mean fluorescence from fluorescence–minus–one controls was subtracted from the sample mean fluorescence to normalize between experimental replicates.

### Monocyte Histone Modification in Response to Immune Training

To assess H3K4 methylation, human monocytes were isolated and adjusted to a density of 1x10^6^ cells/mL in RPMI 1640, and 2 x10^5^ monocytes were plated per well in a 96–well plate. Wells were treated with 10 μg/mL β–glucan or PBS. Glucan was washed out after 24h on day 1, and media was replaced with RPMI 1640 supplemented with 10% human serum. The media was replenished at day 3. On day 7, cells were assayed for H3K4 methylation using the EpiQuik *In Situ* Histone H3K4 Methylation Assay Kit (EpiGentek P–3015–096). To assess H3K27 acetylation, 1 x10^6^ human monocytes were plated in a 12–well plate at 0.5 x 10^5^ cell/mL in RPMI 1640 as previously described. Wells were treated with 10 μg/mL β–glucan or PBS. Glucan was washed out after 24 hrs. on day 1, and media was replaced with RPMI 1640 supplemented with 10% human serum. The media was replenished at day 3. On day 7, histones were isolated using the EpiQuik Total Histone Extraction Kit (EpiGentek OP–0006–100) and frozen at –80°C. H3K27 acetylation was assayed using the EpiQuik Global Acetyl Histone H3K27 Quantification Kit (EpiGentek P–4059–96).

### Statistical Analysis

All statistical analysis was performed in GraphPad Prism 9 or R. All data are presented as individual values or mean ± SEM represented by error bars unless otherwise specified. The effect of age and immune training was evaluated using a mixed–effect model in which all treatment conditions of a blood sample were paired. Šídák’s multiple comparisons test was used for all *post hoc* tests. When the assumption of constant variance was not met, a natural log transformation of the data was performed. Simple linear regression was used to determine the effect of age on metabolic rates and cytokine production. Effect of sex on metabolic rates and cytokine production were assessed using a PERMANOVA because the assumption of constant variance was not met. The effect of trained immunity in sepsis was analyzed using a paired T–test. When the assumption of constant variance was not met, a Wilcoxon test was performed. An α = 0.05 was used to determine the statistical significance of all measurements.

### Study Approval

This study was approved by the East Tennessee State University Institutional Review board (IRB study # 098–98s–MSHA). Written informed consent was obtained prior to participation of patients in this study.

## Results

### Increased Monocyte Metabolism in Response to Immune Training

**Figure 2:**
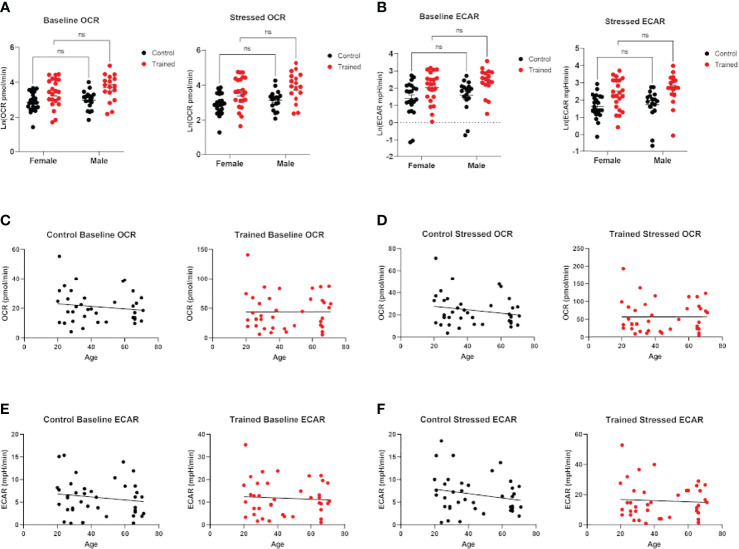
Effect of age and gender on monocyte metabolism in the presence and absence of β–glucan induced immune training. **(A)** Scatter plot of baseline and stressed oxygen consumption rate (OCR) 7 days after β–glucan or PBS treatment (Female: N=22, Male: N=17). **(B)** Baseline extracellular acidification rate (ECAR) and stressed ECAR 7 days after β–glucan or PBS treatment (Female: N=22, Male: N=17). **(C–F)** Simple linear regression of baseline and stressed OCR and ECAR. No correlations are significant (N=13). **(A, B)** were analyzed PERMANOVA for sex and training. ns, not significant.

Cellular metabolism plays a significant role in immune cell function (27–29). Decreases in respiration and glycolysis have been linked to declining immune systems in aging individuals (27, 30, 31). We found that immune training with β–glucan increased baseline and stressed OCR in each age group (**Figure 1B**). Immune training also significantly increased baseline and stressed ECAR in all three age groups (**Figure 1C**). There was no significant difference in the metabolic status of control and immune trained monocytes across the age groups. We also examined the effect of immune training on monocyte metabolism as a function of gender (**Figures 2A, B**) or age (**Figures 2C, D**). We observed no significant correlation as a function of age or gender (**Figures 2A–F**).

### Differential Effect of Monocyte Immune Training on Cytokine Production

**Figure 3:**
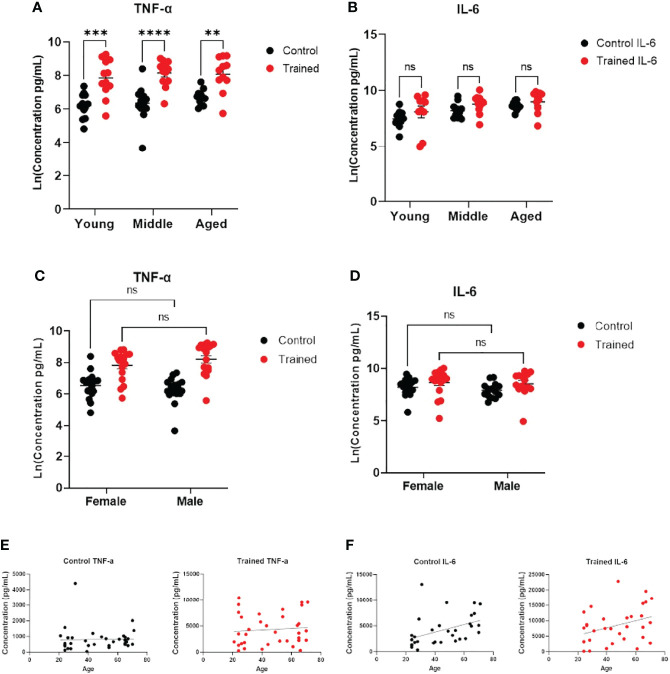
Immune training stimulated increased monocyte cytokine expression. **(A)** Scatter plot of TNF–α expression 7 days after β–glucan or PBS treatment, followed by LPS (N=11,12). TNF–α expression was below the limit of detection in trained and control monocytes that were not subsequently challenged with LPS. **(B)** IL–6 expression (N=10–12). IL–6 expression was limit of detection at baseline. **(C)** Scatter plot of TNF–α expression after LPS stimulation (10ng/mL) 7 days after β–glucan or PBS treatment (Female: N=18, Male: N=14). **(D)** IL–6 expression after LPS stimulation (Female: N=17, Male: N=14). **(E)** Scatter plot and simple linear regression of TNF–α expression by age in control and trained cells. Correlations are not significant (N=35). **(F)** Scatter plot and simple linear regression of IL–6 expression in trained and control cells. Control cells: R^2 =^ 0.1792, P=0.0158 (N=32). Trained cells: R^2 =^ 0.0.1203, P=0.0518 (N=32). A and B measured were analyzed with a mixed effect model and Šídák’s multiple comparisons test. C and D were analyzed by PERMANOVA for sex and training (**P ≤ 0.01, ***P ≤ 0.001, ****P ≤ 0.0001, ns, not significant).

Increased cytokine production in response to immune training with β–glucan is a hallmark of the immune trained phenotype (18, 32). To assess monocyte function, we measured IL–6 and TNF–α secretion following immune training. We found that immune training with β–glucan increased TNF–α expression in all three age groups (**Figure 3A**). There was no significant effect of gender or age on TNF–α expression (**Figures 3C, E**). There was no effect of immune training on IL–6 expression across all age groups. (**Figure 3B**), indicating a differential effect of immune training on cytokine expression. However, IL–6 expression positively correlated with age in untrained monocytes (**Figure 3F**). While there was a trend toward increased IL–6 production as a function of age in trained monocytes (p = 0.0518), it did not reach statistical significance (**Figure 3F**). There was no significant effect of gender on IL–6 expression (**Figure 3D**).

### Differential Effect of Immune Training on Monocyte Histone Methylation and Acetylation

**Figure 4:**
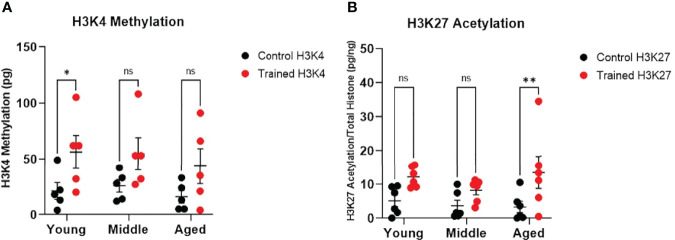
Immune training differentially stimulates monocyte histone modifications. **(A)** H3K4 methylation measured *in situ* 7 days after immune training with β–glucan or baseline control (N=5). **(B)** Histone H3K27 methylation 7 days after immune training with β–glucan or baseline control (N=6). All measured were analyzed with a mixed effect model and Šídák’s multiple comparisons test (*P ≤ 0.05, **P ≤ 0.01, ns, not significant).

Increased H3K4 methylation and H3K27 acetylation are associated with immune training and improved response to infection (18). We found that immune training significantly altered monocyte H3K4 methylation (**Figure 4A**). However, only H3K4 methylation in the 20–30 age group was significantly increased (**Figure 4A**). We also observed that immune training had a significant effect on H3K27 acetylation (**Figure 4B**). Interestingly, H3K27 acetylation was significantly increased in immune–trained cells in the 60+ age group, but not in the younger groups (**Figure 4B**). Thus, we observed a differential effect of β–glucan induced histone modification in that H3K4 methylation was increased in monocytes derived from healthy young individuals, while H3K27 acetylation was significantly increased in monocytes from aging individuals.

### Activation of Intracellular Signaling Pathways in Response to Immune Training

**Figure 5:**
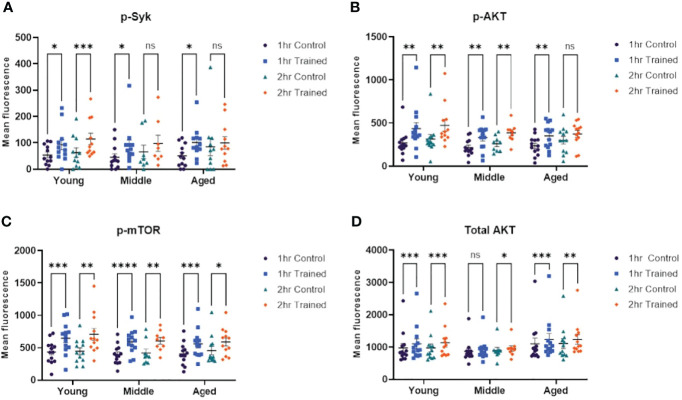
Immune training activates intracellular signaling pathways in monocytes. **(A)** Scatterplot depicting phosphorylated–Syk 1 and 2 hours after β–glucan or PBS treatment (N=8–12). **(B)** Scatterplot depicting phosphorylated–AKT expression (N=9–13). **(C)** Scatterplot depicting phosphorylated–mTOR (N=9–13). **(D)** Scatterplot depicting total AKT expression (N=9–13). All results analyzed using a mixed–effect model and Šídák’s multiple comparisons test (*P ≤ 0.05, **P ≤ 0.01, ***P ≤ 0.001, ****P ≤ 0.0001, ns, not significant).

Cheng and colleagues have shown that β–glucan induced immune training in monocytes is mediated *via* an Akt/mTOR/HIF1–α dependent mechanism (14). Dectin–1 is one of the primary receptors for β–glucan (33, 34) and may be required for β–glucan mediated trained immunity (14). Syk tyrosine kinase is an important downstream mediator of Dectin–1 dependent signaling and is critical in Dectin–1 mediated immune responses (35). We examined the effect of immune training, as a function of age, on Syk, Akt and mTor dependent signaling. We found that phosphorylated Syk was increased in monocytes treated for 1 hour with β–glucan across all age groups (**Figure 5A**). However, only trained monocytes from young individuals expressed significantly higher levels of phosphorylated Syk at 2 hrs. stimulation (**Figure 5A**). We found that AKT phosphorylation was upregulated in monocytes treated for 1 hour with β–glucan across all age groups (**Figure 5B**). Monocytes from the 20–30 and 31–59 age groups that were trained with β–glucan for 2 hrs. showed significantly higher phosphorylated AKT (**Figure 5B**). However, this effect was not observed in the 60+ age group. Phosphorylated mTOR was significantly increased in monocytes treated with β–glucan for one or two hours across all age groups (**Figure 5C**). Total AKT expression was significantly increased in β–glucan trained monocytes at 1 and 2 hrs., although there was no significant difference at 1 hr. in the 31–59 group (**Figure 5D**). The flow cytometry gating strategy for the intracellular signaling studies is shown in **Figures S1, S2**.

### Effect of Immune Training on Monocyte Phenotype as a Function of Age

**Figure 6:**
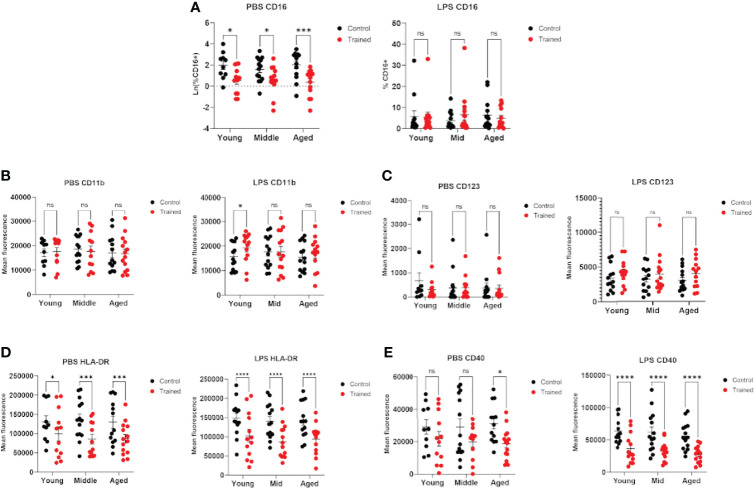
Immune training with β–glucan alters monocyte phenotype. Isolated human monocytes were trained with β–glucan or vehicle control and allowed to rest for 7 days. After 7 days, monocytes were treated with LPS (10ng/mL) or PBS (baseline control) for 24 hours. Cell surface markers were analyzed by flow cytometry. **(A)** Scatterplot depicting the percent of isolated CD14+ monocytes expressing CD16 in LPS–stimulated and unstimulated cells. **(B)** Mean fluorescence of CD11b/Mac–1. **(C)** Mean fluorescence of cell surface CD123. **(D)** Mean fluorescence of cell surface HLA–DR. **(E)** Mean fluorescence of cell surface CD40. All results analyzed using a mixed effect model and Šídák’s multiple comparisons test (*P ≤ 0.05, ***P ≤ 0.001, ****P ≤ 0.0001) (N=10–14). ns, not significant.

Previous studies have demonstrated that age plays a significant role in the phenotype of CD14+ monocytes (36–38). In addition, immune training with β–glucan has been reported to alter the expression of cell surface markers on monocytes (32). We measured the expression of cell surface markers in trained and control monocytes in the presence and absence of LPS secondary stimulation. We found that immune–trained cells in all age groups showed lower levels of CD16+ cells, indicating a higher relative abundance of intermediate or non–classical monocytes (CD14+, CD16+) (39). Interestingly, this effect was not observed after LPS stimulation (**Figure 6A**). CD11b/Mac–1 expression was only significantly altered in trained monocytes from the 20 – 30 age group and only following LPS stimulation (**Figure 6B**). CD123 expression was not altered in immune trained monocytes (**Figure 6C**). In contrast, immune training significantly decreased monocyte cell surface HLA–DR (**Figure 6D**). CD40 was significantly decreased by immune training in monocytes from the 60+ age group (**Figure 6E**). In the presence of LPS secondary stimulation, CD40 was decreased in all three age groups (**Figure 6E**). When taken together, these data are suggestive of differentiation toward an M2 macrophage phenotype.

### β–Glucan Induces the Immune Trained Phenotype in Monocytes Derived From Sepsis Patients

**Figure 7:**
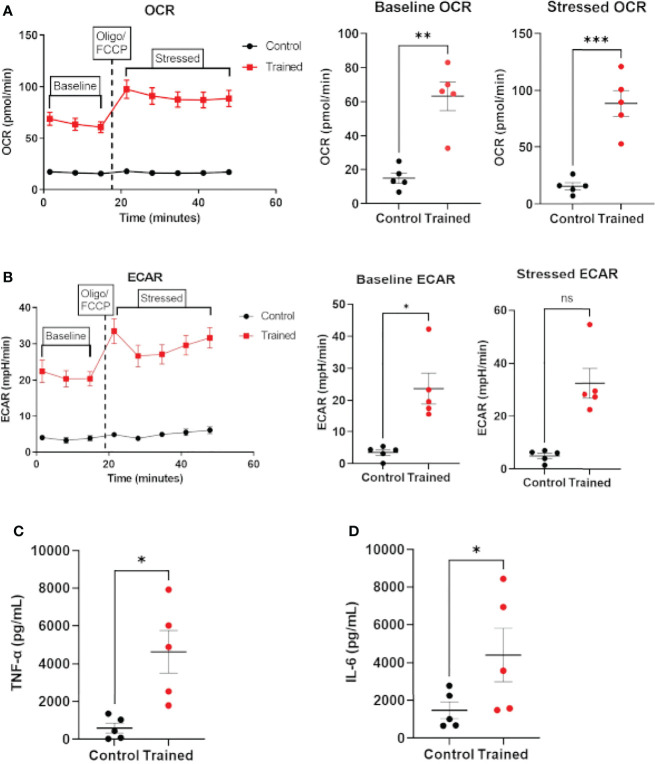
Immune training with β–glucan induces metabolic changes and cytokine expression in monocytes from sepsis patients. **(A)** Baseline and stressed OCR 7 days after β–glucan or PBS treatment. **(B)** Baseline and stressed ECAR 7 days after β–glucan or PBS treatment. Stressed ECAR in trained septic monocytes showed an increased trend versus control monocytes, but did not achieve statistical significance (P=0.0625, N=5). **(C)** TNF–α expression 7 days after β–glucan or PBS treatment after 24h LPS stimulation. **(D)** IL–6 expression. TNF–α and IL–6 expression were below limit of detection levels in cells that were not treated with LPS 7 days after training or control treatment. All measures, unless otherwise specified, were analyzed using a paired t–test (*P ≤ 0.05, **P ≤ 0.01, ***P ≤ 0.001) (N=5). ns, not significant.

The data above clearly show that monocytes from healthy aging and younger individuals can be trained in response to β–glucan. However, there is no data available on the ability of monocytes from septic patients to exhibit the immune trained phenotype. Therefore, we isolated monocytes from patients meeting the broad criteria of Sepsis–3. Monocytes isolated from septic patients were trained with β–glucan employing the protocol in **Figure 1A** and allowed to rest for 7 days. After 7 days, monocytes were assayed for OCR to approximate respiration and ECAR to approximate glycolysis using the Agilent XFp Cell Energy Phenotype Test protocol. Trained septic monocytes had significantly higher baseline and stressed OCR than control septic monocytes (**Figure 7A**). Baseline ECAR was increased in trained septic monocytes (**Figure 7B**). While stressed ECAR showed an increase, there was no statistically significant effect of immune training on stressed ECAR (**Figure 7B**). Additionally, immune trained monocytes from sepsis patients upregulated TNF–α (**Figure 7C**) and IL–6 (**Figure 7D**) upon LPS stimulation. These data clearly show that monocytes from septic patients are capable of exhibiting the immune trained phenotype and their functionality is increased, in a fashion similar to healthy aging subjects.

## Discussion

In the present study, we utilized an *ex vivo* approach to demonstrate that innate immune training can be induced in aging immune cells. We employed β–glucan to induce the immune trained phenotype (40, 41). We found that monocytes from healthy subjects >60 years old display the trained immunity phenotype after exposure to β–glucan. Immune–trained monocytes in aging individuals showed higher metabolic capacities, increased cytokine production, and increased expression of epigenetic markers associated with the trained immunity phenotype (18). In addition, we also found that monocytes from sepsis patients displayed the immune trained phenotype following β–glucan exposure. To the best of our knowledge, this is the first demonstration of innate immune training in human aging and sepsis.

Increased cytokine expression in monocytes following exposure to low levels of LPS is a *ex vivo* hallmark of the trained immune phenotype (14, 24). We observed an increase in TNFα expression in response to immune training similar to other reports (14). However, we did not observe a significant increase in IL–6 in immune trained monocytes as a function of age or gender. This indicates a differential effect of β–glucan induced trained immunity on human monocyte cytokine expression in our model. This result is in contrast to the data of Cheng and colleagues who reported up regulation of TNFα and IL–6 in human monocytes following immune training with the β–glucan training reagent (14).

In addition to characterizing the trained immunity phenotype in β–glucan treated monocytes from aging individuals, we also examined the intracellular signaling pathways associated with the trained phenotype in aging monocytes. Previous studies have shown that Dectin–1 signaling, Akt and mTOR activation are essential for induction of the immune trained phenotype and increased resistance to infection (14). In this study, we observed increased Syk phosphorylation after β–glucan exposure which is consistent with Dectin–1 signaling (34). We also found up–regulation of total AKT, p–AKT, and p–mTOR, which supports activation of these signaling pathways in monocytes across all age groups. There was no effect of age on the expression levels of these key signaling molecules. When taken together these results indicate that pathways crucial to the induction of trained immunity remain viable in healthy aging monocytes and that these pathways can be modulated by β–glucan induced immune training.

Interestingly, immune training led to a relative reduction in CD14^+^/CD16^+^ cells. This population of monocytes is known to be expanded in elderly individuals and is thought to represent a less differentiated population of cells (42). Additionally, we found that immune training decreased expression of the activation markers HLA–DR and CD40 regardless of age. Decreased expression of HLA–DR and costimulatory molecules is suggestive of differentiation toward an M2 macrophage phenotype (43). This result is supported by the study of Leonhardt et al., which found that β–glucan induced monocyte differentiation towards an M2 phenotype (43). Furthermore, this may help to explain the upregulation of the anti–inflammatory cytokine, IL–10, in trained immunity which has been previously described (41), since IL–10 expression is a hallmark of the M2 macrophage. This result suggests that trained macrophages may also play a role in limiting host injury during infection (43).

It is well established that as humans age our immune systems become progressively weaker (1, 2). This unavoidable process begins during the sixth decade of life when the immune system undergoes age related changes which ultimately progress to immunosenescence (1, 2). The impact of immunosenescence is multi–faceted and multi–factorial (1–3). It is also well established that aging is associated with the development of a pro–inflammatory state, known as “inflammaging” (3, 4). In this study, we demonstrated that immune training with β–glucan induced the trained phenotype across all age groups and there was no substantive difference in ability of aging monocytes to exhibit the immune trained phenotype. This finding may have implications for modulation of innate immune function in aging individuals. We also noted that there was a significant correlation between control (non–trained) monocytes derived from healthy aging individuals and increased IL–6 production, which is consistent with age related inflammaging (27). In contrast, we found that immune training diminished monocyte IL–6 production as a function of age. Thus, our data indicate that β–glucan induced immune training did not exacerbate IL–6 production in aging monocytes. Additional studies will be required to further elucidate the impact of immune training on inflammaging.

There are some limitations to our study. We employed an *ex vivo* monocyte model. Thus, we cannot know how immune training would affect other immunocytes or other cells in aging and/or septic individuals. We also did not address how long trained immunity in monocytes may last in this study. This is due to the fact that our *ex vivo* model is not suited for longer–term studies as monocyte viability decreases after 7 days in culture (42, 44). It is also possible that the monocytes isolated in our study differentiated into macrophage–like cells during the seven day incubation. However, we refer to trained and control cells as monocytes due to convention established in previous papers within the trained immunity field (14, 32).

In conclusion, this study confirms that innate immune training can be induced in aging healthy individuals as well as critically ill sepsis patients. We found that innate immune training can be induced regardless of age and there was no substantive difference in the immune trained phenotype as a function of age. We employed β–glucan as our immune training stimulus. The ability of glucan to induce the trained phenotype suggests that it may be possible to pharmacologically induce the immune trained phenotype in aging human immunocytes. When taken together, our data suggests that innate immune training may be a viable prophylactic and/or therapeutic approach to preventing and/or treating infection in vulnerable populations, such as the elderly.

## Data Availability Statement

The raw data supporting the conclusions of this article will be made available by the authors, without undue reservation.

## Ethics Statement

The studies involving human participants were reviewed and approved by East Tennessee State Institutional Review Board. The patients/participants provided their written informed consent to participate in this study.

## Author contributions

PG, TO, ES, and DW conceived, planned and designed the experiments. PG and TO carried out the experiments and acquired the data. PG and NL performed the statistical analyses. PG, TO, NL, ES and DW contributed to data analysis and interpretation of the results. PG took the lead in writing the manuscript. All authors provided critical feedback and helped to shape the final manuscript. All authors contributed to the article and approved the submitted version.

## Funding

This work was supported, in part, by the National Institutes of Health (NIH) Grants R01GM122934 to TO, RO1 AI151210 to consistency, R01GM119197 to ES and DW, RO1GM083016 to DW and C0RR036551 to ETSU. The funding agency had no role in the study design, data collection, data interpretation or preparation of this manuscript. Content of this manuscript has previously appeared online in P. Spencer Gill’s doctoral dissertation “Trained Immunity Enhances the Immune Response and Maintains Microbiome Diversity in Aging and Sepsis.”

## Conflict of Interest

The authors declare that the research was conducted in the absence of any commercial or financial relationships that could be construed as a potential conflict of interest.

## Publisher’s Note

All claims expressed in this article are solely those of the authors and do not necessarily represent those of their affiliated organizations, or those of the publisher, the editors and the reviewers. Any product that may be evaluated in this article, or claim that may be made by its manufacturer, is not guaranteed or endorsed by the publisher.
