# Supplementary material for: Trained Immunity Enhances Human Monocyte Function in Aging and Sepsis
Source: Front Immunol. 2022 May 25;13:872652. doi: 10.3389/fimmu.2022.872652 (PMC9174537; doi:10.3389/fimmu.2022.872652)
Supplement: Supplementary file 1 [file DataSheet_1.pdf]

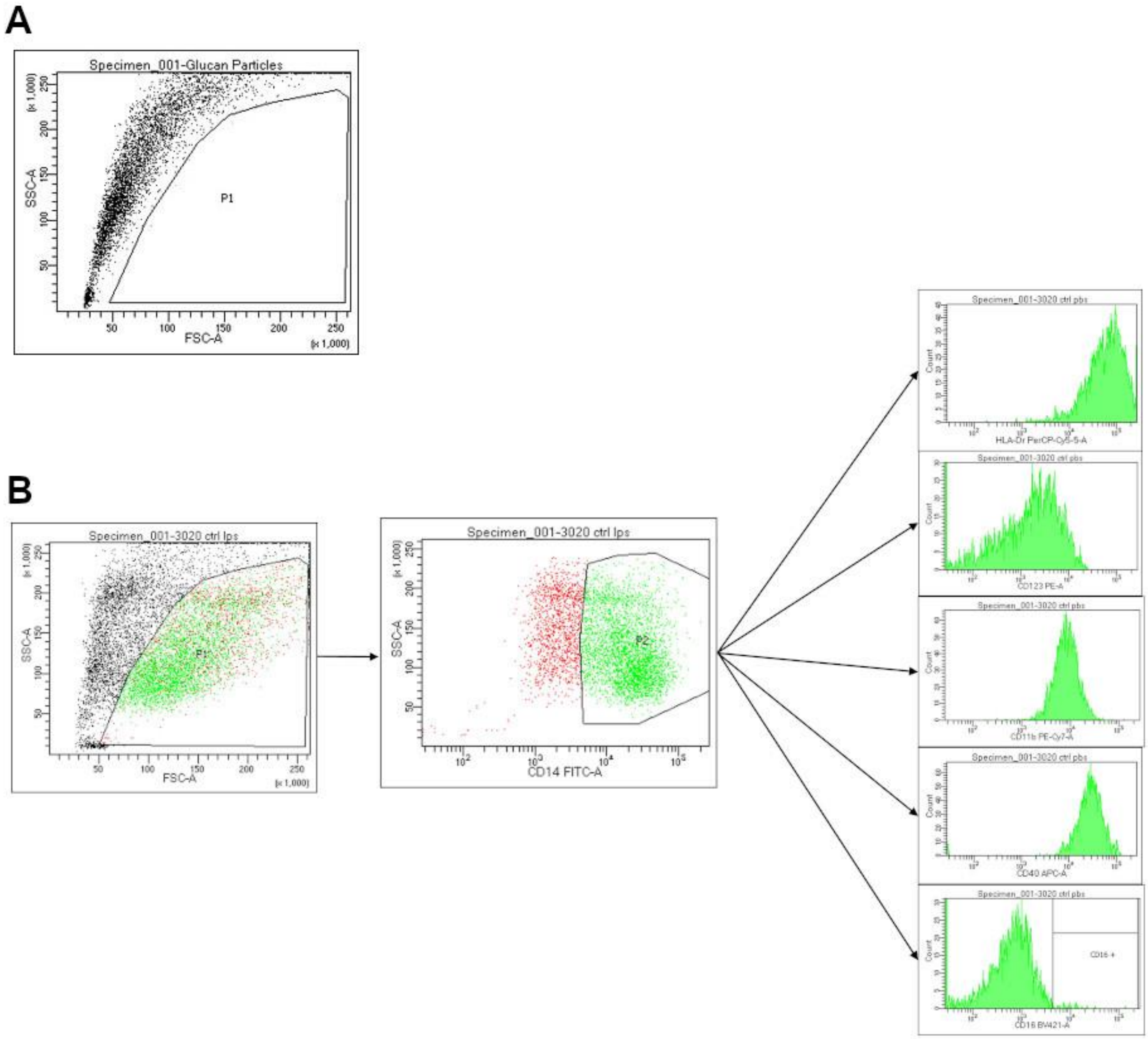

Supplemental Figure 1. Flow cytometry gating strategy for cell surface phenotype experiments. (A) P1 population was set to exclude residual β-glucan particles that could not be removed in wash steps. **(B)** After excluding β-glucan particles, cells were gated for CD14 positivity using fluorescence minus one and isotype controls. Mean fluorescence was used to measure HLA-DR, CD123, CD11b/Mac-1, and CD40. CD16 positive cells were also measured, and gaiting for positivity was determined using FMO and isotype controls.

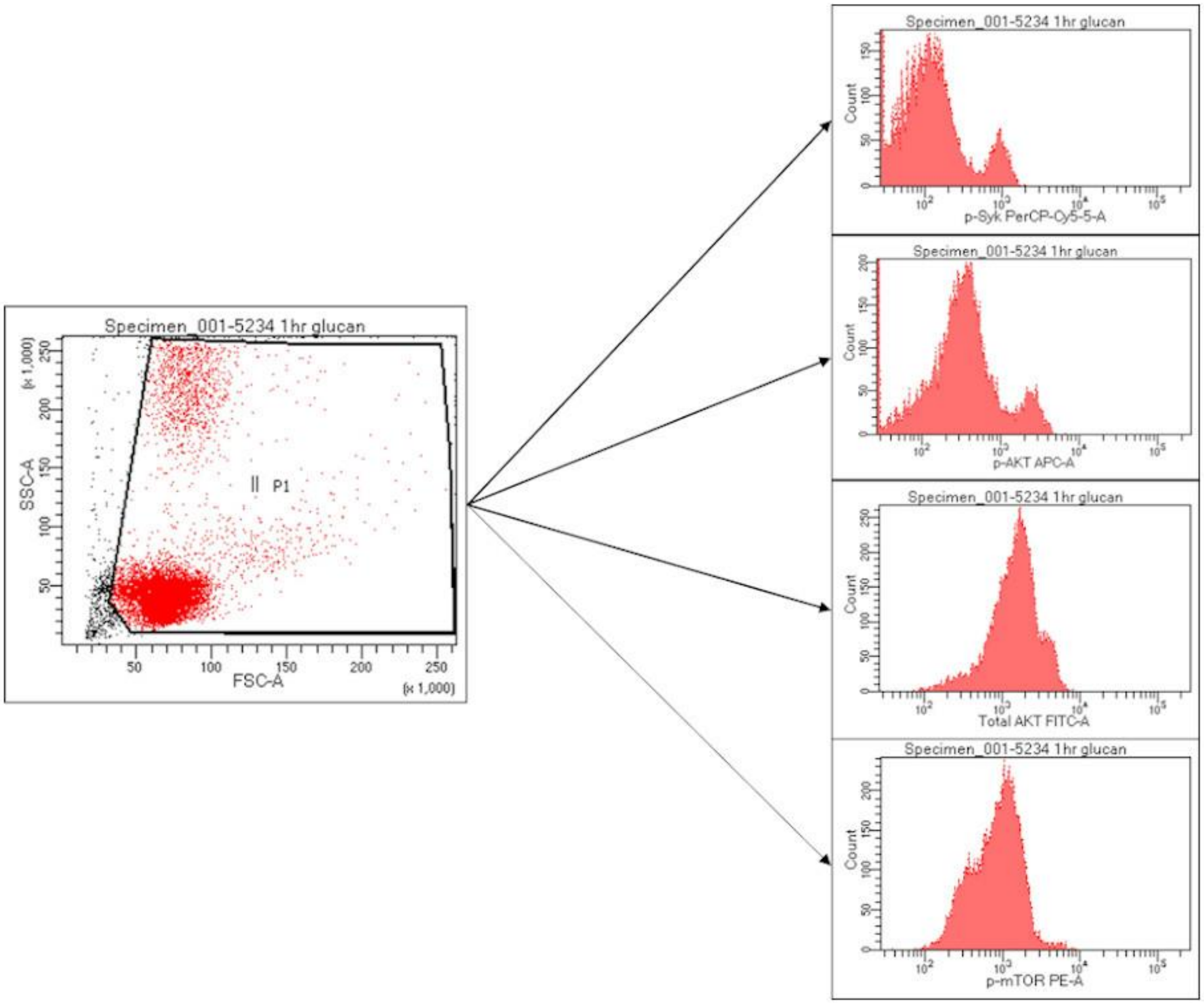

Supplemental Figure 2. Flow cytometry gating strategy for intracellular signaling experiments. Cells isolated using magnetic separation for CD14+ monocytes were gated by FSC and SCC to remove debris. Mean fluorescence of intracellular signaling proteins in this population was used as an experimental measure.
